# Increased knowledge of *Francisella* genus diversity highlights the benefits of optimised DNA-based assays

**DOI:** 10.1186/1471-2180-12-220

**Published:** 2012-09-25

**Authors:** Jon Ahlinder, Caroline Öhrman, Kerstin Svensson, Petter Lindgren, Anders Johansson, Mats Forsman, Pär Larsson, Andreas Sjödin

**Affiliations:** 1 Division of CBRN Security and Defence, FOI, Swedish Defence Research Agency, SE- 906 21, Umeå, Sweden; 2 Department of Clinical Microbiology, Umeå University, SE–901 85, Umeå, Sweden; 3 Laboratory for Molecular Infection Medicine Sweden (MIMS), Umeå University, SE–901 87, Umeå, Sweden

**Keywords:** Bacterial-typing techniques, Optimisation, Francisella, Metagenomics, Phylogeny, Assay, Diversity, NGS, PCR

## Abstract

**Background:**

Recent advances in sequencing technologies offer promising tools for generating large numbers of genomes, larger typing databases and improved mapping of environmental bacterial diversity. However, DNA-based methods for the detection of *Francisella* were developed with limited knowledge about genetic diversity. This, together with the high sequence identity between several *Francisella* species, means there is a high risk of false identification and detection of the highly virulent pathogen *Francisella tularensis*. Moreover, phylogenetic reconstructions using single or limited numbers of marker sequences often result in incorrect tree topologies and inferred evolutionary distances. The recent growth in publicly accessible whole-genome sequences now allows evaluation of published genetic markers to determine optimal combinations of markers that minimise both time and laboratory costs.

**Results:**

In the present study, we evaluated 38 previously published DNA markers and the corresponding PCR primers against 42 genomes representing the currently known diversity of the genus *Francisella*. The results highlight that PCR assays for *Francisella tularensis* are often complicated by low specificity, resulting in a high probability of false positives. A method to select a set of one to seven markers for obtaining optimal phylogenetic resolution or diagnostic accuracy is presented.

**Conclusions:**

Current multiple-locus sequence-typing systems and detection assays of *Francisella*, could be improved by redesigning some of the primers and reselecting typing markers. The use of only a few optimally selected sequence-typing markers allows construction of phylogenetic topologies with almost the same accuracy as topologies based on whole-genome sequences.

## Background

The gram-negative pathogen *Francisella tularensis* is the causative agent of tularemia and is classified as a category-A biological-threat agent [1]. Natural transmission of tularemia to humans is complex, occurring via the inhalation of infective aerosols, ingestion of contaminated water, handling sick or dead animals, ingestion of infected food-stuffs, or bites of infected arthropods such as ticks, biting flies or mosquitoes [2].

The genus *Francisella* includes a number of closely related but ecologically distinct species that can be divided into two main genetic clades [3]. These bacteria exhibit a large variety of lifestyles, including specialised intracellular pathogens of mammals (*F. tularensis* subsp. *tularensis* and subsp. *holarctica*) and fish (*F. noatunensis*), *Francisella*-like endosymbionts (FLEs) (represented here by *Wolbachia persica*) and freely living generalists (*F. philomiragia* x *F. novicida*) causing disease predominantly in humans with a compromised immune defense [4]. The taxonomic boundaries of *Francisella* have recently been debated, in particular for *F. novicida*[5,6]. Recent breakthroughs in sequencing techniques have enabled public access to whole-genome sequences that can shed light on previously unknown diversity within the *Francisella* genus. The mode of genetic inheritance varies within the genus: the overall recombination rate is 34% of the genes within the *Francisella* core genome, although recombination is virtually non-existent in *F. tularensis* and *F. noatunensis*[3,7]. These ecological and reproductive differences which lead to genetic diversity make *Francisella* an ideal choice for evaluation of diagnostic PCR-based DNA markers and developing sample sequencing methods for phylogenetic analyses.

Over the last decade, PCR methods have been successfully applied for the rapid identification and classification of *Francisella* isolates [8]. An obvious drawback with DNA-based approaches is the possibility of cross-reactivity with non-pathogenic but closely related *Francisella* subspecies occurring naturally in the environment [3,9,10]. This could distract biological surveillance systems, such as the BioWatch Program [11], and give false-positive alarms [12,13]. Therefore, primer pairs need to be defined so that an unknown isolate is identified and attributed to the correct species or subspecies. Previously published sequence markers designed for identification or detection of *Francisella* have been developed without taking into consideration the current knowledge of genetic diversity of the genus, in particular the recently discovered species *F. noatunensis* and *F. hispaniensis*.

The specificity of *Francisella* detection assays has often been controlled by testing reactivity with non-*Francisella* bacterial species. Typically, no other species besides *F. tularensis* (including subspecies *tularensis, mediasiatica* and *holarctica*), *F. novicida* and *F. philomiragia* have been included as representatives of the *Francisella* genus [14-17]*.* As with PCR detection, current knowledge on the diversity of the *Francisella* genus affects the choice of genetic markers used for obtaining true phylogenetic trees by PCR-based sequence-typing analysis. For *F. tularensis*, multi-locus typing schemes targeting overlapping, as well as separate, genes have been described [18,19]. However, the resolution was limited, allowing discrimination of only the major genetic clades of the species. Recent advances in sequencing and the increased availability of publicly accessible genomic sequences have enabled phylogenetic trees obtained by analysing sequence markers to be evaluated. Whole-genome sequencing is not always desirable for large bacterial sample sets, as such analysis normally generates large amount of data which requires substantial increase in labour and time. Therefore, multiplexed target amplification of selected genomic regions using next generation sequencing (NGS) have recently been proposed [20,21].

A considerable effort in the study of bacterial pathogens has been devoted to evaluating alternative evolutionary histories by comparing topologies [22-25]. In order to facilitate these comparisons, various topological distance metrics have been proposed, such as the Robinson-Foulds (RF) or symmetric distance [26], branch-score distance [27], path-length metrics [28] and nearest-neighbour interchanging [29]. To quantify similarity, all these metrics focus on topological features (order of nodes within the topology) and/or branch- or path-length differences (between nodes and leaves). An alternative approach would be to construct and test a parameter describing the degree of incompatibility (i.e. conflicting phylogenetic signals) between topologies. To the best of our knowledge, no such straightforward metric exists for this particular purpose of quantifying the level of incompatibility. Alternative topologies could be compared with a reference topology obtained from, e.g. the literature, a large set of concatenated genes or a source of high-quality whole-genome data. Ideally, such reference topology should mimic the species phylogeny as accurate as possible.

In this study, we evaluated the specificity of detection and classification of *Francisella* by first comparing published PCR primers against whole-genome sequences representing the known diversity of the genus. Second, we examined the sequence-marker robustness and resolution by comparing different sets of one to seven markers using a modified version of the RF metric. Finally, we showed that optimal sets of markers outperform other combinations with respect to phylogenetic robustness and resolution.

## Results

### Overall fit between DNA-markers and whole-genome sequences of *Francisella*

**Figure 1:**
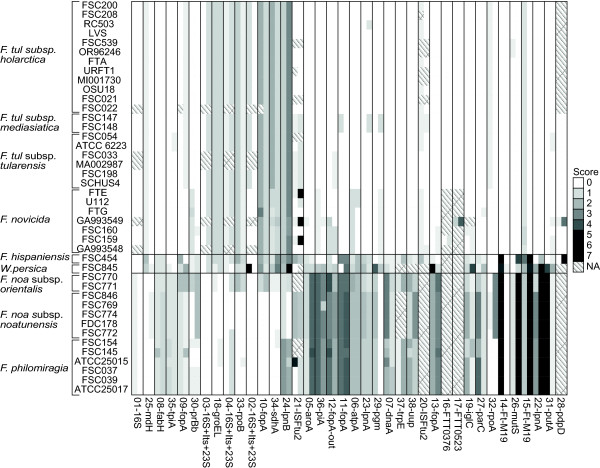
**Overview of primer specificity.** Weighted score of primer specificity calculated with penalties for mismatches and gaps, where zero indicates a perfect match. The first column of each marker represents the forward primer score and the second represents the reverse primer score. The score was calculated with PrimerProspector as follows: 3’ mismatch, 1 penalty per mismatch (length of 3’ region was set to 5), non-3’ mismatch, (0.4 penalty per mismatch), last base mismatch (penalty 3 per mismatch), non 3’ gap (penalty 1 per gap) and 3’ gap (penalty 3 per gap).

A total of 42 publicly available *Francisella* genome sequences were screened for sequences (Table 1) of 38 published markers (Table 2). 14 markers had incomplete sets of marker sequences (Figure 1). The lack of 16S marker sequences in FSC022, FSC033, MA002987, GA993549, and GA993548 was probably due to the low quality of the genome sequences, which were all sequenced with early versions of 454 sequencing technology. The lack of sequences for the remaining 10 markers was most likely because they were designed for real-time PCR molecular detection or possibly due to uncovered regions in the sequence (Additional file 1).

**Table 1:** Genomes sequences included in the study

**Species**	**ID**	**BioProject ID**
*F. tularensis subsp. holarctica*	FSC200	16087
*F. tularensis subsp. holarctica*	FSC208	73467
*F. tularensis subsp. holarctica*	RC503	30637
*F. tularensis subsp. holarctica*	LVS	16421
*F. tularensis subsp. holarctica*	FSC539	73393
*F. tularensis subsp. holarctica*	OR96-246	30669
*F. tularensis subsp. holarctica*	FTA	20197
*F. tularensis subsp. holarctica*	URFT1	19645
*F. tularensis subsp. holarctica*	MI00-1730	30635
*F. tularensis subsp. holarctica*	OSU18	17265
*F. tularensis subsp. holarctica*	FSC021	73369
*F. tularensis subsp. holarctica*	FSC022	19015
*F. tularensis subsp. mediasiatica*	FSC147	19571
*F. tularensis subsp. mediasiatica*	FSC148	73379
*F. tularensis subsp. tularensis*	FSC054	73375
*F. tularensis subsp. tularensis*	ATCC6223	30629
*F. tularensis subsp. tularensis*	FSC033	19017
*F. tularensis subsp. tularensis*	MA00-2987	30443
*F. tularensis subsp. tularensis*	FSC198	17375
*F. tularensis subsp. tularensis*	SCHUS4 (FSC237)	9
*F. novicida*	FTE	30119
*F. novicida*	U112	16088
*F. novicida*	FTG	30447
*F. novicida*	GA99-3549	19019
*F. novicida*	FSC160	73385
*F. novicida*	FSC159	73383
*F. novicida*	GA99-3548	19573
*F. hispaniensis*	FSC454	73391
*Wolbachia persica*	FSC845	73171
*F. noatunensis subsp. orientalis*	FSC770	73389
*F. noatunensis subsp. orientalis*	FSC771	73447
*F. noatunensis subsp. noatunensis*	FSC846	73463
*F. noatunensis subsp. noatunensis*	FSC769	73397
*F. noatunensis subsp. noatunensis*	FSC774	73457
*F. noatunensis subsp. noatunensis*	FDC178	73465
*F. noatunensis subsp. noatunensis*	FSC772	73449
*F. philomiragia*	FSC154	73381
*F. philomiragia*	FSC145	73377
*F. philomiragia*	ATCC25015	32411
*F. philomiragia*	FSC037	73371
*F. philomiragia*	FSC039	73373
*F. philomiragia*	ATCC25017	27853

Francisella genomes included in this study selected to represent the known diversity of *Francisella*: 22 strains representing the public health perspective of *F. tularensis* (clade 1) and 13 strains of *F. noatunensis* and *F. philomiragia* (clade 2) representing a fish farming industry and health perspective.

**Table 2:** **A list of the markers selected to represent published DNA-based markers for molecular PCR detection or phylogenetic identification targeting *Francisella***

**Marker name/ Target gene**	**Gene locus_tag**^**a**^	**Amplicon size (bp)**^**a**^	**Genomic location**^**a**^	**Reference**
01-16S	FTT_r04, FTT_r07, FTT_r10	1139	1311156-2294, 1378275–9413, 1771610-2748	[17,37,38,56]
02-16 s + ItS + 23 s	FTT_r04, FTT_r07, FTT_r10	915	1311470-2371, 1378876–9490, 1771911-2825	[34]
03-16 s + ItS + 23 s	FTT_r03-FTT_r04, FTT_r06-FTT_r07, FTT_r09-FTT_r10	948	1310519-1466, 1377638–8585, 1770973-1920	[34]
04-16 s + ItS + 23 s	FTT_r03, FTT_r06, FTT_r09	925	1309613-10537, 1376732–7656, 1770067-991	[34]
05-aroA	FTT_0588	650	608150-799	[18,61]
06-atpA	FTT_0062	634	62762-3395	[18,61]
07-dnaA	FTT_0001	618	303-920	[19]
08-fabH	FTT_1373	1289	1418892-20155	[62]
09-fopA	FTT_0583	886	599105-990	[19]
10-fopA	FTT_0583	1068	599148-600215	[34]
11-fopA-in	FTT_0583	404	599526-929	[15]
12-fopA-out	FTT_0583	708	599428-600135	[15]
13-fopA	FTT_0583	86	599767-852	[9,16]
14-FtM19	FTT_1472c	250	1524132-381	[56,58]
15-FtM19	FTT_1472c	316	1524066-381	[65]
16-FTT0376	FTT_0376	107	377718-824	[17]
17-FTT0523	FTT_0523	91	546620.712	[17]
18-groEL	FTT_1696	803	1764659-5461	[34]
19-iglC	FTT_1712c	84	1792514-597	[9,16]
20-ISFtu2^b^	FTT_1311	390	1335726-6115	[56,59]
21-ISFtu2	FTT_0099^c^	97	103438-534^c^	[9,16]
22-lpnA^b^	FTT_0901	407	909857-10263	[19,37,38,56,57]
23-lpnA	FTT_0901	93	910211-301	[9,16]
24-lpnB	FTT_0904	252	911795-2046	[34]
25-mdh	FTT_0535c	715	556932-7646	[63,64]
26-mutS	FTT_1499	495	1553224-3718	[19]
27-parC	FTT_0396	643	397063-705	[18,61]
28-pdpD	FTT_1360c, FTT_1715c	136	1403503-638, 1796838-973	[56,60]
29-pgm	FTT_0414	650	425033-682	[18,61]
30-prfB	FTT_0191	376	207686-8061	[19]
31-putA	FTT_1150c	415	1165411-825	[19]
32-rpoA	FTT_0350, FTT_1442c	914	349619-50532	[64]
33-rpoB	FTT_0144	262	156309-570	[34]
34-sdhA	FTT_0074	223	75065-287	[34]
35-tpiA	FTT_0080	484	83679-4162	[19]
36-tpiA	FTT_0080	559	83657-4215	[18,61]
37-trpE	FTT_1802c	517	1888928-9444	[18,61]
38-uup	FTT_0445	645	459229-873	[18,61]

^**a**^ Amplicon locus tag, length and location in genome of F. tularensis strain SCHU S4.

^b^ Primer sequence of primer Tuf1705 in marker 20-ISFtu2 and TUL-435 in marker 22-lpnA seem to be incorrectly specified in [56]. See [37] and [59] for the correct primer sequences.

^c^ Insertion element present in multiple copies in reference. Only first position and gene specified.

The primer specificities of the 38 DNA markers were calculated, resulting in scores ranging from 0 to 7.2 (Figure 1). Importantly, the calculation was performed for *Francisella* species besides those included in the publication from which the marker originated. A primer score of zero represented a perfect match without any mispriming events or gaps, while the maximal score of 7.2 corresponded to two mismatches in the 3’ region and a gap of 10 bases within the region targeted by a primer (see marker 21-ISFtu2). All primer scores are presented in Figure 1 and summarised in Table 2. The limit for possible amplification was assumed to be a score value of two, in agreement with the NCBI Primer-BLAST default primer specificity stringency setting. Scores below two (<2) are denoted as low score and score above two (≥2) are denoted as high score [30].

### Evaluation of DNA markers

The marker 01-16S [14] targeting 16S rRNA was the only marker with a low score (<1) for all the investigated genomes. A total of nine markers (01-16S, 03-16S-Itr-23S, 04-16S-Itr-23S, 08-fabH, 18-groEL 23-lpnA, 25-mdh, 30-prfb and 35-tpiA) had scores < 2 in all subspecies. However, some of these markers, e.g. 23-lpnA, showed a clear difference in scores between clade 1 and clade 2, as clade 1 yielded almost perfect matches, while scores in clade 2 were always > 1.

Most of the included primers amplified sequences of *F. tularensis* (including subspecies *tularensis, mediasiatica,* and *holarctica*) and *F. novicida* of clade 1 and less frequently amplified sequences of *F. noatunensis* and *F. philomiragia*, of clade 2. Fifteen markers (05-aroA, 07-dnaA, 11-fopA-in, 12-fopA-out, 13-fopA, 14-FtM19, 15-FtM19, 19-iglC, 22-lpnA, 26-mutS, 27-parC, 31-putA, 36-tpiA, 37-trpE and 38-uup) gave low scores for clade 1 and high scores for clade 2. Marker 38-uup also had low scores in one isolate of *philomiragia*, and the marker 19-iglC had low scores in *F. noatunensis* subsp. *orientalis* and in two isolates of *F. philomiragia*.

Of these fifteen markers, twelve (05-aroA, 07-dnaA, 12-fopA-out, 13-fopA, 19-iglC, 22-lpnA, 26-mutS, 27-parC, 31-putA, 36-tpiA, 37-trpE and 38-uup) had low scores for *F. hispaniensis* FSC454 and/or *W. persica* FSC845 as well as low scores in clade 1. Only three (11-fopA-in, 14-Ft-M19 and 15-Ft-M19) out of the fifteen markers consistently differentiated clade 1 from the rest of the *Francisella* genus.

The marker 10-fopA was the only marker completely specific for clade 2 and only marker 24-lpnB was specific for *F. noatunensis*. Both of these exhibited lower specificity for *F. noatunensis* subsp. *orientalis* genomes.

Several markers displayed complex amplification patterns. Seven markers (02-16S-Itr-23S, 06-atpA, 09-fopA, 29-pgm, 32-rpoA, 33-rpoB, 34-sdhA) had high scores in one or more species or subspecies, e.g. the marker 09-fopA had a low score in all included strains except in *F. hispaniensis* FSC454 and *W. persica* FSC845. Similar results were observed for 02-16S-Itr-23S, 29-pgm, 33-rpoB and 34-sdhA.

Four detection markers (16-FTT0376, 17-FTT0523, 20-ISFtu2 and 28-pdpD) had missing data (i.e. the sequence could not be found in the genome) for all clade 2 isolates plus *W. persica*. The markers 16-FTT0376 and 17-FTT0523 had missing sequences for *F. hispaniensis* and *F. tularensis* subsp. *novicida*, except the isolates FSC159 and GA993549, respectively. The marker 21-ISFtu2 had missing sequences as well as mismatches in almost all subspecies represented. A summary of the DNA-marker evaluation can be found in Table 3, and more detailed information, including earlier published results for each marker, can be found in Additional file 1.

**Table 3:** **Summary of estimated amplification performance of primer pairs representing published DNA-based markers targeting *Francisella***

**Estimated amplification performance**	**Marker id**
**Amplifies the entire genus**	01-16S, 03-16S-Itr-23S, 04-16S-Itr-23S, 08-fabH, 18-groEL, 23-lpnA^a^, 25-mdh, 30-prfb and 35-tpiA.
**Amplifies clade 1 but not clade 2**	05-aroA, 07-dnaA, 11-fopA-ina^a^, 12-fopA-out^a^, 13-fopA^a^, 14-FTM19^b^, 15-FTM19, 19-iglC^ac^, 22-lpnA^a^, 26-mutS, 27-parC^c^, 31-putA, 36-tpiA, 37-trpE and 38-uup.
Amplifies clade 1 but no other *Francisella* species.	11-fopA-in^a^, 14-FtM19 and 15-FtM19^a^
Amplifies clade 1 as well as *F. hispaniensis* and *W. persica*	05-aroA, 07-dnaA, 12-fopA-out^a^, 27-parC^c^ and 36-tpiA.
Amplifies clade 1 as well as *F. hispaniensis*	13-fopA^a^, 19-iglC^c^, 22-lpnA, 31-putA, 37-trpE and 38-uup.
Amplifies clade 1 as well as *W. persica*	26-mutS
**Amplifies clade 2 but not clade 1**	10-fopA
**Amplifies noatunensis but not the other species**	24-lpnB
**Amplifies all isolates except some certain species.**	02-16S-Itr-23S, 06-atpA, 09-fopA, 29-pgm, 32-rpoA, 33-rpoB and 34-sdhA.
Amplifies all except *F. hispaniensis* and *W. persica*	09-fopA
Amplifies all except *F. hispaniensis*	33-rpoB
Amplifies all except *F. tularensis*, *W. persica* and *F. hispaniensis*	34-sdhA
Amplifies all except *W. persica*	02-16S-Itr-23S, 29-pgm
Amplifies all except *F. noatunensis* subsp. *orientalis*	06-atpA
Amplifies all except *F. noatunensis*	32-rpoA
**Markers with data missing for clade 2 and W. persica**	16-FTT0376^a^, 17-FTT0523^a^, 20-ISFtu2^b^ and 28-pdpD^b^.
Amplifies only *F. tularensis* (only when including the probe).	16-FTT0376^a^ and 17-FTT0523^a^
Amplifies *F. tularensis* subsp. *mediasiatica*, *F. tularensis* subsp.*holarctica* and 6/7 *F. tularensis* subsp. *novicida.*	28-pdpD^b^
Amplifies isolates from all clade 1 species as well as *W. persica*.	20-ISFtu2^b^
**Marker with missing sequences as well as mismatches in almost all subspecies represented.**	21-ISFtu2^a^

Successful amplification was defined as having a primer score below two in both the forward and reverse primers.

^a^ Have associated TaqMan probe which is not considered here. ^b^Detection by variable-length amplicon which is not considered here.

^c^Score of *F.noatunensis* subsp *orientalis* <2.

### Evaluation of sample-sequencing approaches for phylogenetic analyses

**Figure 2:**
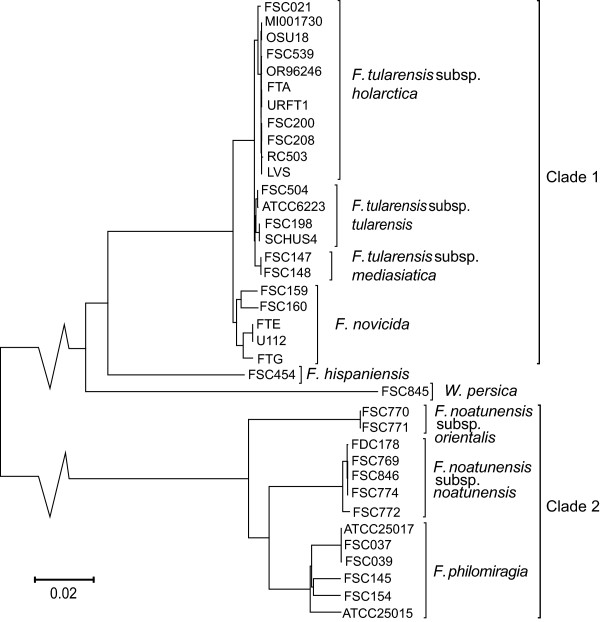
**Whole-genome SNP phylogeny.** The whole-genome phylogeny for 37 *Francisella* strains obtained with model averaging implemented in jModelTest using PhyML software. The removed part of the branches connecting clade 1 and 2 covers a genetic distance of 0.03.

**Figure 3:**
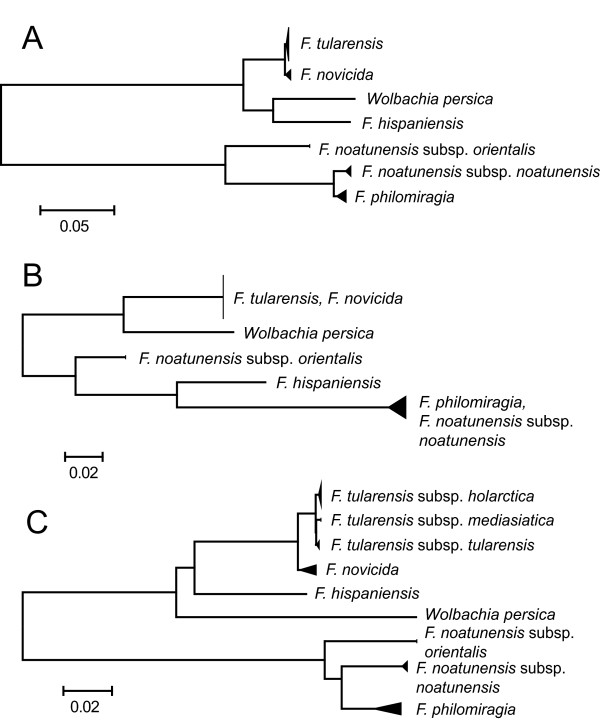
**Single-marker phylogenies.** Single-marker phylogeny of the *Francisella* genus: (**A**) highest ranked marker 08-fabH, (**B**) lowest ranked marker 33-rpoB, and (**C**) whole-genome phylogeny. Rank is based on difference in resolution between alternative and whole-genome topology.

In the phylogenetic comparison analysis, we focused not only on the entire *Francisella* genus, but also analysed clades 1 and 2 separately. These sub-populations exhibit different lifestyles and environmental niches and are therefore of interest to different scientific fields [3,7,18]. The differences between the poorest and best resolved single marker topologies of the entire genus compared to the whole-genome reference topology (Figure 2) are highlighted in Figure 3A-C. All topologies are shown in Additional File 2. The parameter estimates of the phylogenetic analysis are summarised in Additional File 3. In general for the analysis of the entire genus, the optimal substitution model was parameter rich, i.e. typically the generalised time-reversible (GTR) [31] or Hasegawa-Kishino-Yano (HKY85) [32] models with either invariant sites parameter (*α*) or rate heterogeneity over sites (*Г*). Moderate or even low parameter-rich substitution models were favoured in the separate clade analyses, in particular for clade 1, where Jukes-Cantor (JC) [33] or HKY85 models were found to be the optimal choice without *α* or *Г*. For clade 2, it was important to include the proportion of invariant sites parameter in the analyses, because of detected recombination events [3].

Throughout the study, to facilitate the phylogeny comparisons, we made use of two metrics: degree of incongruence (inc) and difference in resolution (res). The two topologies compared were the reference topology, obtained from whole genome data, and the single-sequence or the concatenated marker sequences topology. Results from comparing single-sequence topologies against the reference phylogeny based on whole-genome sequences are summarised in Additional File 4. The comparisons varied in inc, and sometimes considerably so. In the analysis of the entire genus, the 37-trpE topology did not exhibit any incongruence compared to the reference (inc = 0), although the resolution was poor. For other markers, such as 08-fabH, 27-parC, 03-16 s + ItS + 23 s, 04-16 s + ItS + 23 s, 25-mutS and 36-tpiA, the topology comparisons indicated few mismatched bipartitions (inc < 0.25), whereas the opposite result was found for 11-fopA-in, 29-pgm and 30-prfB (inc > 0.35). As expected, for some single-marker topologies, particularly those with the lowest inc scores, the SH test did not reject congruence compared to the reference phylogeny.

Separate clade 1 topologies exhibited a lower average incongruence than topologies of the entire genus (inc_clade1_ = 0.139 vs. inc_genus_ = 0.258, *p* = 6.6e-05) and clade 2 topologies (inc_clade1_ = 0.139 vs. inc_clade2_ = 0.238, *p* = 0.0149). In several cases, clade 1 topologies were totally congruent with no mismatched bipartitions. Some of these topologies were also congruent in clade 2: (01-16S, 03-16 s + ItS + 23 s, 04-16 s + ItS + 23 s, 07-dnaA, 08-fabH, 22-lpnA, 24-lpnB, 25-mdh, 27-parC, 30-prfB, 31-putA, 35-tpiA, 36-tpiA, 37-trpE and 38-uup). The low level of incongruence was verified by the results of the SH-test, which showed that congruence in the topology comparisons could not be rejected with the exception of 19-iglC. Reported incongruences in clade 1 mostly occurred in *F. novicida*. Most assignments deviating from the reference in clade 2 were due to misplacements of subspecies *F. philomiragia* and *F. noatunensis* subsp*. noatunensis*.

In the separate analysis of clade 1, most strains not assigned according to the reference were due to poor resolution, notably topologies of markers 32-rpoA, 37-trpE, 25-mdh, 24-lpnB and 19-iglC. The average resolution (res) in topologies of clade 1 was significantly higher than clade 2 (res_clade1_ = 0.723 vs. res_clade2_ = 0.604, *p* = 0.003) and the entire genus (res_clade1_ = 0.723 vs. res_genus_ = 0.664, *p* = 0.010). The correlations between the incongruence and resolution metrics were *ρ* = 0.405 and *ρ* = 0.484 for clade 1 and 2, respectively.

**Figure 4:**
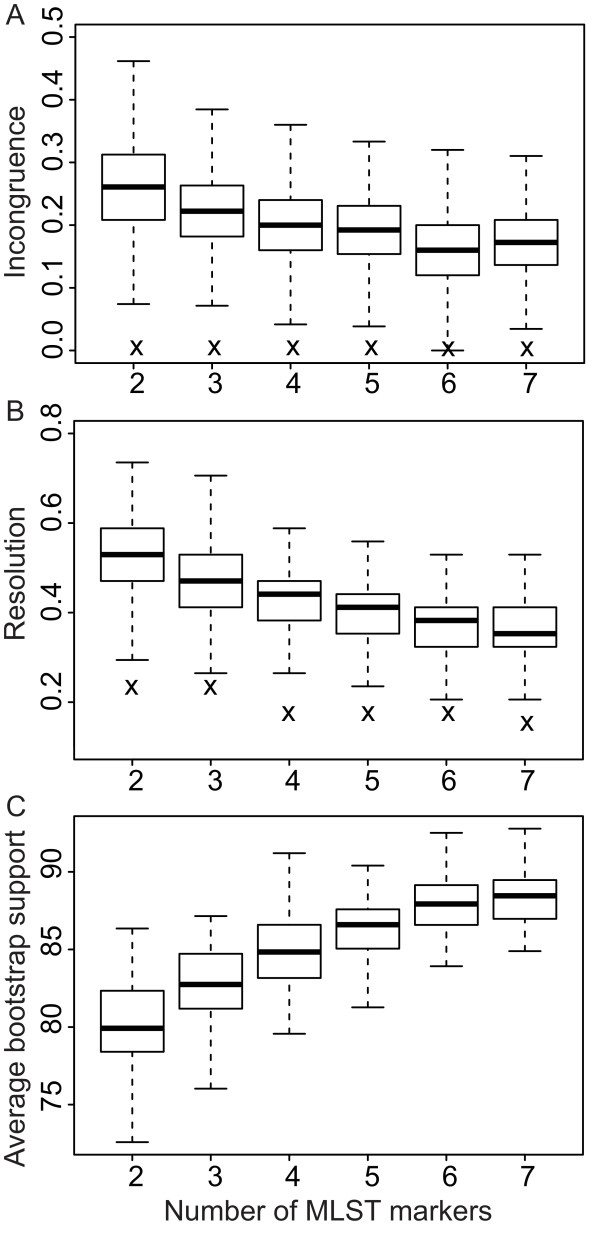
**The impact of the number of markers on phylogenetic parameters.** The effect of concatenating sequence markers on topology (of the *Francisella* genus) in comparison with the whole-genome tree for (**A**) incongruence score, (**B**) resolution score, and (**C**) average bootstrap support from 1000 replicates. The results of the optimised topology comparisons are shown as crosses.

Figure 4 shows the difference in comparison metrics and average bootstrap support (boot) when markers were randomly concatenated and an optimised combination of markers was selected. Table 4 lists optimal sets of two to seven markers for use in studies of the *Francisella* genus. Summary statistics of the optimal combinations of markers in the entire genus are summarised in Additional File 5. Results of the optimisation analyses of the separate clades are not shown. Compared to random concatenation of sequence markers, the *Francisella* genus topology from an optimised set of markers reduced the difference in resolution by on average 50 - 59% and totally eliminated incongruences. The suggested combination of five gene fragments in [34] resulted in a topology comparison with res = 0.471 and inc = 0.217, whereas the corresponding optimal topology resulted in res = 0.176 and inc = 0.000. The average bootstrap support of the optimised topologies compared to the average bootstrap of random marker topologies was significantly higher for congruence at the 5 marker level (boot_opt_ = 88.33 vs. boot_rand_ = 86.38, *p* < 0.001), 6 marker level (boot_opt_ = 88.67 vs. boot_rand_ = 87.81, *p* < 0.001), and 7 marker level (boot_opt_ = 88.92 vs. boot_rand_ = 88.29, *p* < 0.001), as well as for resolution at the 6 marker level (boot_opt_ = 90.71 vs. boot_rand_ = 87.81, *p* < 0.001).

**Table 4:** **Summary of the optimisation procedure for resolution (res) and congruence (inc) in the *Francisella *genus where the consensus set of markers are highlighted according to how often they are selected in the optimal partitions of markers; position 1 corresponds to the most represented marker**

	**Position**	**1**	**2**	**3**	**4**	**5**	**6**	**7**
**No of markers**	**Metric**							
2	res	08-fabH	35-tpiA					
	inc	08-fabH	35-tpiA					
3	res	08-fabH	35-tpiA	24-lpnB				
	inc	08-fabH	35-tpiA	02-16 s				
4	res	08-fabH	35-tpiA	24-lpnB	27-parC			
	inc	35-tpiA	08-fabH	01-16S	02-16 s			
5	res	08-fabH	35-tpiA	24-lpnB	27-parC	22-lpnA		
	inc	35-tpiA	08-fabH	24-lpnB	27-parC	33-rpoB		
6	res	08-fabH	24-lpnB	35-tpiA	27-parC	22-lpnA	25-mdh	
	inc	35-tpiA	08-fabH	24-lpnB	04-16 s	01-16S	33-rpoB	
7	res	08-fabH	35-tpiA	24-lpnB	26-mutS	27-parC	18-groEL	22-lpnA
	inc	35-tpiA	08-fabH	01-16S	04-16 s	24-lpnB	27-parC	25-mdh

Markers 02-16 s + ItS + 23 s and 04-16 s + ItS + 23 s are abbreviated as 02-16 s and 04-16 s, respectively.

## Discussion

Knowledge about theoretical limitations of marker assays is important for the successful detection and identification of bacteria in research as well as public health contexts. Existing methods for detection and identification of *Francisella* were developed with limited knowledge about the genetic diversity within the *Francisella* genus. From a clinical perspective, the lack of knowledge of diversity in the environment may be of minor importance since diagnostic sampling is performed on humans or animals suspected of having the disease. In contrast, use of the same detection assays for environmental sampling can lead to problems with false positive results. The recent increase in publicly available genome sequences enables development of improved detection and identification methods for both purposes. The emergence of high-throughput typing of large collections of bacterial strains targeting single amplicons is likely to mean that the targeting of single-marker regions will continue to be important in the future [20].

In this study, we evaluated 38 published markers (Table 2) against the current known diversity of the *Francisella* genus. It is important to note that the studies from which the markers were gathered differed widely in scope. Some studies were designed to only cover a specific species and exclude others, whereas in other studies it was not of interest or even possible to study all the *Francisella* species included here. Several of the included markers were amplifying sequence products for species not included in previous studies of *Francisella,* e.g. *F. hispaniensis*, *F. noatunensis* and *W. persica*. As many as one third of the markers amplified all the included subspecies and approximately half of the markers amplified products for *F. hispaniensis* and/or *W. persica* together with clade 1 or clade 2. This indicates that strains belonging to *F. hispaniensis*, *W. persica*, *F. noatunensis* are responsible for several false identifications. It should be pointed out that we have only considered sequence based markers here. Other type of markers and marker combinations can be fruitful, in particular for construction of sub-species specific assays, which has been shown by e.g. combining variable-number of tandem repeats (VNTR) and insertion-deletion (indel) markers [35] or SNP and indel markers [36].

Specificity is especially important for markers designed for detection. The results of the investigated detection markers suggested that the specificity was questionable for the majority of them. The marker 22-lpnA [37,38], designated for *F. tularensis* detection, was found to also amplify *F. hispaniensis* FSC454 [39]. In the present study, the primers of the genus-specific marker 13-fopA [16] were not predicted to amplify any of the included *F. philomiragia*, whereas in the original publication they were reported to amplify all included *F. philomiragia* isolates. Probably a large unknown diversity exists within this species. For almost all 11 detection markers for *Francisella tularensis*, there was a significant risk of false-negative results caused by unwanted mismatches for isolates that should be detected. In conclusion, primer sequences need to be continually evaluated and redesigned using up-to date knowledge of the genetic diversity of the targeted sequences to minimise the likelihood of false-positive or -negative results. A similar conclusion was published by [40] where false-positive and -negative hits of primers against publically available sequences in various species of bacteria were evaluated with the result of high degree of primer mismatch in *Haemophilus influenza*, *Pseudomonas aeruginosa* and *Escherichia coli*. Hence, primer miss-match seems to be a general problem within prokaryotes. Our evaluation approach for primers could subsequently be of benefit to the microbiological community.

In order to compare analyses based on PCR-based sequence data with analyses based on whole-genome data for making phylogenetic inferences, we partitioned the popular RF metric into two separate metrics, incompatibility and resolution, to enable comparison of an alternative topology with a reference topology. These two metrics explain different characteristics, which allow a particular question to be considered when evaluating the phylogeny of bacteria given the reference topology. In the genomes of *Francisella* analysed here, these two metrics were correlated and therefore displaying similar metric characteristics, albeit with some exceptions, particularly in the clade 1 analysis. The incompatibility metric was negatively correlated with nucleotide diversity, whereas the opposite was found for the resolution metric, which highlights differences in the characteristics of these metrics. This finding suggests that single-nucleotide polymorphisms (SNPs) in marker-sequence regions increase the resolution but may also compromise the phylogenetic signal. One possible explanation for the incompatibility of SNPs and whole-genome phylogeny is the presence of recombination within sequence fragments, which has been suggested by several previous analyses of pathogenic bacteria populations; i.e. *Neisseria meningitidis*[22,25,41], *Staphylococcus aureus*[22,42] and *Escherichia coli*[22,43]. Nonetheless, for analysis of large numbers of bacterial strains showing conflicting topologies using different combinations of markers, our proposed comparison metrics are useful measures. To determine whether the observed topological differences could have occurred by chance, our comparison approach can be combined with a statistical test, such as the SH test applied here or an alternative test, e.g. [44,45].

Most incompatibilities were associated with the topologies that included all strains, whereas the level of incompatibility was significantly lower for clade 1, with topologies being totally compatible in many cases. These results indicate that the clonal frame is maintained within the *F. tularensis* clade, but it is disrupted at the genus level and in clade 2. Most incompatibilities were a result of *F. philomiragia, F. novicida*, *W. persica* and *F. hispaniensis* strains that were misplaced in the single-marker cases, which suggests that recombination is the main evolutionary force that promotes incongruences in *Francisella*, as pointed out by, e.g. [7,18]. The reduction of recombination rate in clade 1 might, in turn, reflect barriers to gene flow between ecological and geographical clusters among sub-species [7,46-49].

Our result suggests that no single-marker topology of the entire genus is able to assign all strains to the subspecies defined by the whole genome topology. In fact, some marker topologies, such as 02-16 s + ItS + 23 s and 24-rpoB, made deviating assignments in more than 70% of the cases. The reason for the low success rate of assigned strains to their corresponding sub-species is mainly poor resolution, which meant that typically all *F. tularensis* strains displayed identical sequences. Most topologies assigned all strains to the same main clades as in the whole genome phylogeny, with a few exceptions: 33-rpoB assigned *F. hispaniensis* to clade 2 and 19-iglC assigned *W. persica* to clade 2, in subgroup *F. noatunensis* subsp. *orientalis* (in both assignments). This is an interesting observation as rpoB was recently suggested as an alternative marker to 16S rDNA in metagenomic studies [21].

The level of incompatibility and difference in resolution compared to the whole-genome reference topology were decreased, in some cases by a considerable amount, by selecting an optimal combination of markers. Moreover, topologies based on an optimal set of markers significantly increased the average statistical support (i.e. average bootstrap). Generally, both the degree of compatibility and resolution were improved by concatenating sets of two to seven markers in all possible combinations. However, some combinations, in particular considering incompatibility, might result in poorer topologies than for an estimated topology based on a single marker. This observation is consistent with previous work where concatenation of sequence data have resulted in biased phylogenetic estimates [50]. All incompatible phylogenetic signals were removed in topologies based on optimised sets of two to seven markers, in contrast to random concatenation. Totally congruent topologies were obtained by concatenating as few as only two markers (08-fabH and 35-tpiA). These two markers were included in all optimal sets. Hence, by selecting an optimal set of markers, a large improvement in resolution and compatibility can be obtained over random concatenation.

An exhaustive search strategy was employed to find the optimal set of markers since the total number of available markers was relatively small. It should be pointed out that the number of possible marker combinations increases rapidly with the number of markers considered and soon becomes computationally intractable. As all the 742 gene fragments of the core genome in the analysed population have recently become available in [3], an interesting extension to the current work would be to find the optimal set of markers based on all those genes. Such an optimisation could be carried out by utilising one of the myriad of available optimisation techniques, such as a simulated annealing approach [51,52]. It should be noted that we do only try to minimize the value of the objective metrics, incongruence or resolution difference, with respect to the whole-genome topology. There is no guarantee that the whole genome topology accurately resembles the true underlying species topology as systematic errors and statistical inconsistencies in the phylogenetic inference method could be amplified when analyzing whole genome data [50,53-55].

By demonstrating the potential of establishing robust bacterial phylogenies using sample sequencing of only a few markers, we believe that the framework presented here could serve as a foundation for population analyses as well as for identifying and attributing unknown pathogenic strains to the correct subspecies.

## Conclusions

The results of this study suggest that several of the investigated markers designed to be diagnostic exhibit a considerable level of unspecificity. Hence, several of the currently used primers need to be redesigned to avoid false-positive results. This arises because of a previous lack of knowledge about genetic diversity within the *Francisella* genus represented by, e.g. strains belonging to *F. hispaniensis* and among FLEs. By employing sample sequencing of DNA markers to make phylogenetic inferences, we revealed incompatibilities among topologies that included all considered *Francisella* strains but not among topologies that included only clade 1 strains containing *F. tularensis*. An estimated topology based on optimised combination of markers drastically reduced incompatibility and resolution differences compared to topologies obtained by random concatenation and at the same time improved the average bootstrap support, using the whole genome phylogeny as a reference. Implementation of such an optimisation framework based on accurate reference topology would help to improve assays for detection and identification purposes, which are of considerable importance in a number of research fields, such as for improving biosurveillance systems and inferring evolutionary histories.

## Methods

### Bacterial strains

A total of 37 genome sequences (Table 1) were selected to represent the known diversity of *Francisella*. This collection included both pathogenic and non-pathogenic strains and could be divided into two major clades. The public-health perspective was represented by 22 strains of the human pathogen *F. tularensis* (clade 1) and the fish-farming industry and health perspective was represented by 13 strains of *F. noatunensis* and *F. philomiragia*, which are all fish pathogens (clade 2). In addition, the strain *Wolbachia persica* FSC845, representing the FLEs, and the newly discovered *F. hispaniensis* FSC454 were included. More detailed information about the included strains has been published elsewhere [3].

### PCR markers

The study focused on a set of 38 markers used in detection or identification of *Francisella* (Table 2). A subset of 13 markers (01-16S [14,37,38,56], 22-lpnA [19,37,38,56,57], 13-fopA, 19-iglC, 21-ISFtu2, 23-lpnA [9,16], 11-fopA-in, 12-fopA-out [15], 14-FtM19 [56,58], 16-FTT0376, 17-FTT0523 [17], 20-ISFtu2 [56,59] and 28-pdpD [56,60]) were originally designed primarily for real-time PCR molecular detection of *Francisella* at different taxonomic levels; genus, species or subspecies (here called detection markers).

A subset of 25 markers (02-16S + ItS + 23S, 03-16S + ItS + 23S, 04-16S + ItS + 23S, 10-fopA, 18-groEL, 24-lpnB, 33-rpoB, 34-sdhA [34], 05-aroA, 06-atpA, 27-parC, 29-pgm, 36-tpiA, 37-trpE, 38-uup [18,61] 07-dnaA, 09-fopA, 26-mutS, 30-prfB, 31-putA, 35-tpiA [19], 08-fabH [62], 25-mdh [63,64] 32-rpoA [64], 15-FtM19 [65]), which were originally designed for PCR-based identification (here called identification markers), were also included.

The primer specificity was tested for all 38 markers. In the topological comparisons and optimisation procedures, 28, 27 and 26 markers were used for clade 1, clade 2 and the whole-genome data, respectively (see Additional File 1 for details).

### *In silico* PCR

PCR fragments were assumed to result from all included genomes rather than exclusively the genomes considered in developing the marker. An *in silico* PCR fragment was first generated for one selected isolate (*F. tularensis* subsp. *tularensis* SCHU S4, *F. tularensis* subsp. *holarctica* FSC200 or *F. noatunensis* subsp. *noatunensis* FSC769) using multithreaded electronic PCR (mismatches allowed = 4, expected length = 2000 bp, margin = 400 bp, honouring IUPAC ambiguity in STS) [66], which is an enhanced version of electronic PCR [67] . This fragment was then aligned to the rest of the genomes using Exonerate v2.2.0 (model: est2genome, percent threshold = 70, score threshold = 50, maxintron length = 2500) [68]. Finally, all fragments for each marker were aligned using MUSCLE v3.7 using default settings [69].

### PCR-primer scoring

Primer specificity was evaluated by scoring each primer sequence against the corresponding *in silico* generated target sequences using PrimerProspector [70]. To direct the scoring to the region where the primer sequence aligned for all strains, the primer region was extracted from the alignment and used alone as input to the scoring software. The weighted score was calculated based on 3’ mismatch (penalty 1 per mismatch, 3’ length 5), non-3’ mismatch (penalty 0.4 per mismatch), last-base mismatch (penalty 3 per mismatch), non 3’ gap (penalty 1 per gap) and 3’ gap (penalty 3 per gap). The lowest possible score in this type of calculation is zero, which is only achieved when the primer is a perfect match. The score, which is based on mismatches and gaps, is dependent on primer length, and thus a max score cannot be given. The limit for a possible PCR amplification was set to 2, in agreement with the NCBI Primer-BLAST default primer specificity stringency setting for amplification, i.e. at least two mismatches in the 3’ region. According to latter system, scores below two are regarded as low scores, whereas scores greater than or equal to two are regarded as high scores. Calculated scores for forward and reverse primers for each strain were clustered with DIvisive ANAlysis clustering in the cluster package [71] and then plotted in a heatmap using the ggplot2 package [72] in R v2.13.1 [73].

### Phylogenetic analysis

Phylogenetic trees were inferred using two alternative methods: neighbour joining (NJ) [74] and maximum likelihood (ML) [75]. The software packages PhylML 3.0 [76,77] and Phylip [78] were used. In the NJ analysis, 1000 bootstrap replicates were calculated in the software Seqboot and summarised in the Consense software. The genetic distances between strains were estimated with the software Dnadist by employing the F84 nucleotide substitution model [79]. The NJ tree was inferred with the Neighbour software, in the Phylip package [76]. By using the software jModelTest [80], we were able to evaluate alternative nucleotide substitution models for the maximum likelihood analysis and perform model averaging [81], in which the alternative models were weighted based on the fit to the data and model complexity (i.e. the number of effective parameters in each substitution model) using the Bayesian information criterion (BIC) [82]. Substitution models with unequal base frequencies, a proportion of invariable sites, *α*, and allowance for rate variation among sites, *Г*, were included. The number of discrete gamma categories was 4. In total, we considered 24 alternative substitution models in the model-averaging process. The more computationally intense ML procedure was chosen to estimate phylogenies in the single-marker analysis, whereas the rapid NJ method was utilised in the multiple marker analyses. The whole-genome phylogeny was estimated with both the ML and NJ methods by considering 20,072 SNPs on the core genome of all 37 genomes. The SNPs were obtained using the same procedure as in [3], where the Mauve software [83] with default options was used to perform multiple genome alignment and in-house perl-script was used to identify the SNPs based on the obtained alignments. As both ML and NJ methods resulted in virtually identical phylogenies, we concluded that the choice of estimation method did not have a significant impact on the evaluation of the sequence-marker topologies.

### Phylogenetic-topology comparison

To check for and quantify the degree of compatibility between the phylogenetic trees estimated with marker-sequence data and the whole-genome tree (i.e. two trees with nested taxa), bipartitions in the marker tree were checked for their presence/absence in the whole-genome tree. In trees with missing sequences, the corresponding leaves were removed from the whole-genome tree using the R package ape [84]. The output, i.e. number of absent bipartitions, were normalised by the total number of bipartitions in the marker tree. This topology metric was denoted inc throughout the study. For perfectly compatible trees, no bipartitions in the marker tree should be absent in the whole-genome tree. To obtain the bipartitions at the internal edges of the trees, the output from the Consense software in the Phylip package [78], together with an in-house Perl script (available upon request), were used. The inc metric is similar to the RF distance [26], although the RF metric counts the number of bipartitions not present in the other tree for both trees. Therefore, the RF metric measures both the degree of incongruence and the difference in resolution between reference and alternative topologies. By modifying the RF distance metric, the degree of incongruence can be quantified more precisely and also separated from the difference in resolution between the compared topologies. In a similar manner, a Perl script was implemented to count the number of bipartitions present in the whole-genome topology that were absent in the alternative topology (i.e. difference in resolution, denoted res) and to normalise the output to vary between 0 and 1. As a reference, RF distances (also known as symmetric differences) implemented in the Treedist software [78] were used. To investigate the success of the marker tree to allocate a strain to its corresponding sub-species family (according to the whole genome phylogeny), bipartition scoring in the Consense software was used and the output was compared to the pre-defined subspecies bipartitions according to the whole-genome tree. In addition, we investigated whether strains were assigned to the corresponding main clades of the entire *Francisella* genus, reporting the proportion of misidentified strains on each clade. Finally, we considered the average bootstrap support of each marker tree.

It is important to consider a statistical test for topological incongruence as stochastic effects in the evolution of the sequences results in incongruence between the compared trees. To address this issue, we employed the Shimodaira-Hasegawa (SH) test [85], which is a non-parametric test for determining whether there are significant differences between conflicting topologies in specific sequence data. The null hypothesis of the SH test assumed that the compared topologies were equally probable given the data. Here, we tested the marker topologies and the whole-genome topology on each respective marker sequence using the phyML software package by fixing the topologies and optimising the substitution model and branch-length parameters. The SH test was performed within the CONSEL software package [86], which takes the output from phyML as input. Since multifurcations in topologies are strongly penalised in the phyML software, we resolved the topologies into bifurcating trees using the R package ape [84]. The substitution model selected in the phyML analysis was based on the preferred substitution model of the jModelTest analysis. To test whether clades differed in incongruence or resolution, a Wilcoxon rank sum test with continuity correction was utilised, implemented in the R statistical package [73]. We used Spearman’s rank correlation coefficient, *ρ*, to quantify correlations between metrics and the average pairwise nucleotide diversity, *π*, of the clades.

### Optimisation procedure

Since the number of included sequence markers in this study was moderate, we searched through all possible combinations of markers (i.e. an exhaustive search). We performed two separate analyses, one for each of the metrics used: incongruence and difference in resolution between topologies. The marker configuration(s) resulting in the lowest metric value were saved. The code was written in Perl and is available upon request from JA.

To test whether the average bootstrap support obtained from optimised topologies and topologies generated by random concatenation differed, we again made use of the Wilcoxon rank sum test with continuity correction in cases where more than 10 optima were found. The null hypothesis was that the level of average bootstrap support was equivalent for the optimised and randomised topologies. Due to the high computational demands, we only analysed 100 topologies obtained by random concatenation of sequences with respect to bootstrap support. Furthermore, we compared the optimal topology identified here to the topology obtained by analysing the sequence combination suggested by [34]: 33-rpoB, 10-fopA, 18-groEL, 24-lpnB and 34-sdhA.

## Abbreviations

BIC: Bayesian information criterion; Clade 1: Population including *F. tularensis* subspecies; Clade 2: Population including *F. noatunensis* subspecies and *F. philomiragia*; Entire genus: Entire genus all included strains representing all known subspecies; FLEs: *Francisella* like endosymbionts; GTR: Generalised time reversible; HKY85: Hasegawa-Kishino-Yano; Indel: Insertion-deletion; JC: Jukes Cantor; ML: Maximum likelihood; NGS: Next generation sequencing; NJ: Neighbour joining; RF: Robinson-Foulds; SH: Shimodaira-Hasegawa; SNP: Single-nucleotide polymorphisms; VNTR: Variable number of tandem repeats.

## Authors’ contributions

JA and CÖ wrote script code, extracted and analysed the data; JA, CÖ, and AS wrote the manuscript; KS, PLI, AJ, MF, PLA contributed to writing the manuscript; AJ, MF, PLA and AS organised and conceived the study. All authors read and approved the final manuscript.

## Supplementary Material

Additional file 1 **Summary of earlier published and current results of investigated sequence markers.** A list of earlier published as well as current results of the specificity of each marker at subspecies level, presence/absence of the markers in the different clades, details of which parts of the study the marker was included and marker type. Click here for file

Additional file 2 **Single-marker topologies.** A zip-file containing all single-marker topologies in pdf format obtained from the model-averaging phylogenetic analysis using jModelTest. Click here for file

Additional file 3 **Parameter estimates obtained from the phylogenetic analysis.** Summary statistics of the single-marker phylogenetic analysis. The most optimal DNA substitution model was selected by BIC implemented in jModelTest. Standard errors of average bootstrap supports are shown in parentheses. The estimated proportion of invariable sites is the expected frequency of sites that do not evolve. Click here for file

Additional file 4 **Table of single-marker results.** Comparison of inferred single-gene topologies to the whole-genome topology with respect to RF distance degree of incongruence, difference in resolution, the proportion of misidentified strains and SH test of incongruence. To test alternative topologies for markers with missing sequences, the corresponding leaves were removed from the whole-genome tree. Click here for file

Additional file 5 **Optimal set of marker partitions.** Optimisation of the subset of two to seven marker-sequence topologies to minimise incongruences and difference in resolution compared to the whole-genome topology. The numbers show the percentage of each marker included in the optimal configurations. The proportion of strains misplaced in the tree, average bootstrap support of optimal topologies and the SH test of incongruence is also reported. The total number of global optima was calculated from the output of the heuristic search analyses. Click here for file
