# Supplementary material for: Increased knowledge of Francisella genus diversity highlights the benefits of optimised DNA-based assays
Source: BMC Microbiol. 2012 Sep 25;12:220. doi: 10.1186/1471-2180-12-220 (PMC3575276; doi:10.1186/1471-2180-12-220)
Supplement: Additional file 3 — Parameter estimates obtained from the phylogenetic analysis. Summary statistics of the single-marker phylogenetic analysis. The most optimal DNA substitution model was selected by BIC implemented in jModelTest. Standard errors of average bootstrap supports are shown in parentheses. The estimated proportion of invariable sites is the expected frequency of sites that do not evolve. [file 1471-2180-12-220-S3.docx]

## Additional File 3. Summary statistics of the single marker phylogenetic analysis.

Reported estimates are model averaged over 24 alternative substitution models, implemented in the jModelTest software. Most optimal DNA substitution model was selected by the Bayesian Information Criterion (BIC). The estimated proportion of invariable sites is the expected frequency of sites that do not evolve. The analysed populations are denoted F, CL1 (clade 1) and CL2 (clade 2) for the *Francisella* population, for the *Francisella tularensis* subpopulation and for the *Francisella noatunensis* and *Francisella philomiragia*, respectively.

| Marker | Data | Total number of bases | Prop. of invar. sites | α | Ts/Tv | f(AT) | Pref. subst. model |
| --- | --- | --- | --- | --- | --- | --- | --- |
| 01-16S | F | 1140 | 0.930 | 0.027 | 3.218 | 0.487 | HKY+I |
|  | CL1 | 1140 | 0.922 | 62.19 | 1714 | 0.482 | HKY |
|  | CL2 | 1140 | 0.971 | 0.033 | 20.25 | 0.487 | HKY+I |
| 02-16s+ItS+23s-1 | F | 922 | 0.908 | 0.027 | 3.127 | 0.489 | HKY+I |
|  | CL1 | 916 | 0.884 | 62.19 | 3.000 | 0.482 | F81 |
|  | CL2 | 916 | 0.959 | 0.032 | 10.69 | 0.488 | HKY+I |
| 03-16s+ItS+23s-2 | F | 954 | 0.813 | 0.037 | 2.935 | 0.525 | K80+I |
|  | CL1 | 949 | 0.802 | 62.19 | 5.005 | 0.515 | JC |
|  | CL2 | 941 | 0.154 | 90.14 | 8.035 | 0.516 | K80 |
| 04-16s+ItS+23s-3 | F | 650 | 0.531 | 0.351 | 2.314 | 0.654 | HKY+I |
|  | CL1 | 925 | 0.969 | 0.014 | 7.856 | 0.535 | HKY |
|  | CL2 | 925 | 0.515 | 62.19 | 9.066 | 0.535 | HKY |
| 05-aroA-2 | F | 650 | 0.531 | 0.351 | 2.314 | 0.654 | HKY+I |
|  | CL1 | 650 | 0.003 | 90.04 | 2.002 | 0.633 | HKY |
|  | CL2 | 650 | 0.772 | 0.034 | 1.581 | 0.658 | HKY+I |
| 06-atpA | F | 635 | 0.696 | 0.141 | 2.835 | 0.633 | GTR+G |
|  | CL1 | 635 | 0.929 | 0.014 | 10.13 | 0.601 | HKY |
|  | CL2 | 634 | 0.850 | 0.031 | 4.136 | 0.633 | HKY+I |
| 07-dnaA | F | 621 | 0.648 | 0.193 | 2.148 | 0.705 | HKY+I |
|  | CL1 | 618 | 0.810 | 0.119 | 2.417 | 0.688 | HKY |
|  | CL2 | 618 | 0.801 | 0.036 | 3.810 | 0.690 | HKY+I |
| 08-fabH | F | 1310 | 0.626 | 0.205 | 2.895 | 0.682 | HKY+G |
|  | CL1 | 1290 | 0.001 | 90.46 | 6.684 | 0.665 | HKY |
|  | CL2 | 1296 | 0.766 | 0.033 | 3.610 | 0.665 | HKY+I |
| 09-fopA-1 | F | 886 | 0.544 | 0.338 | 1.949 | 0.652 | GTR+I |
|  | CL1 | 886 | 0.078 | 90.45 | 8.024 | 0.633 | HKY |
|  | CL2 | 871 | 0.721 | 0.146 | 2.627 | 0.646 | HKY+G |
| 10-fopA-2 | F | 1068 | 0.534 | 0.371 | 1.734 | 0.649 | GTR+G |
|  | CL1 | 1068 | 0.003 | 90.03 | 5.270 | 0.633 | HKY |
|  | CL2 | 1053 | 0.748 | 0.110 | 2.927 | 0.647 | HKY+G |
| 11-fopA-in | F | 404 | 0.581 | 0.304 | 1.970 | 0.585 | GTR+I |
|  | CL1 | 404 | 0.750 | 62.19 | 3.005 | 0.579 | JC |
|  | CL2 | 404 | 0.745 | 0.112 | 2.237 | 0.585 | HKY |
| 12-fopA-out | F | 708 | 0.575 | 0.310 | 1.983 | 0.611 | GTR+I |
|  | CL1 | 708 | 0.481 | 62.19 | 4.012 | 0.591 | HKY |
|  | CL2 | 702 | 0.859 | 0.033 | 4.358 | 0.614 | HKY+I |
| 18-groEL | F | 803 | 0.709 | 0.125 | 2.759 | 0.641 | GTR+G |
|  | CL1 | 803 | 0.941 | 0.012 | 5.400 | 0.626 | HKY+I |
|  | CL2 | 803 | 0.759 | 0.044 | 2.472 | 0.636 | HKY+I |
| 19-iglC | F | 84 | 0.670 | 0.114 | 1.526 | 0.628 | K80+I |
|  | CL1 | 84 | 0.702 | 62.19 | 2035 | 0.571 | JC |
|  | CL2 | 84 | 0.749 | 0.073 | 2.207 | 0.607 | K80+I |
| 22-lpnA | F | 418 | 0.400 | 0.659 | 1.989 | 0.665 | HKY+G |
|  | CL1 | 408 | 0.937 | 0.014 | 6.304 | 0.656 | HKY+I |
|  | CL2 | 395 | 0.753 | 0.129 | 4.475 | 0.649 | HKY |
| 24-lpnB | F | 255 | 0.586 | 0.210 | 2.662 | 0.683 | HKY+G |
|  | CL1 | 252 | 0.069 | 62.19 | 6.016 | 0.648 | HKY |
|  | CL2 | 255 | 0.810 | 0.027 | 4.541 | 0.657 | HKY+I |
| 25-mdh | F | 715 | 0.589 | 0.279 | 2.109 | 0.621 | GTR+I |
|  | CL1 | 715 | 0.793 | 62.19 | 10.07 | 0.627 | HKY |
|  | CL2 | 715 | 0.001 | 95.16 | 2.683 | 0.617 | HKY |
| 26-mutS | F | 496 | 0.589 | 0.279 | 0.000 | 0.621 | GTR+I |
|  | CL1 | 496 | 0.701 | 0.266 | 4.118 | 0.645 | HKY |
|  | CL2 | 495 | 0.600 | 0.371 | 17.20 | 0.657 | HKY |
| 27-parC | F | 643 | 0.591 | 0.265 | 3.111 | 0.671 | HKY+G |
|  | CL1 | 643 | 0.917 | 0.015 | 4.226 | 0.665 | HKY+I |
|  | CL2 | 643 | 0.815 | 0.029 | 3.277 | 0.666 | HKY+I |
| 29-pgm | F | 651 | 0.576 | 0.257 | 2.306 | 0.648 | HKY+G |
|  | CL1 | 651 | 0.782 | 0.135 | 2.339 | 0.631 | HKY+G |
|  | CL2 | 650 | 0.715 | 0.130 | 7.127 | 0.623 | HKY+I |
| 30-prfB | F | 376 | 0.506 | 0.452 | 2.450 | 0.602 | HKY+G |
|  | CL1 | 376 | 0.003 | 89.31 | 3.346 | 0.604 | HKY |
|  | CL2 | 376 | 0.708 | 0.149 | 2.868 | 0.603 | HKY |
| 31-putA | F | 415 | 0.635 | 0.210 | 3.607 | 0.684 | HKY+G |
|  | CL1 | 415 | 0.2277 | 62.19 | 1.665 | 0.6464 | F81 |
|  | CL2 | 415 | 0.718 | 0.174 | 3.712 | 0.683 | HKY |
| 32-rpoA | F | 920 | 0.598 | 0.267 | 2.747 | 0.678 | HKY+I |
|  | CL1 | 914 | 0.970 | 0.014 | 1.256 | 0.663 | F81 |
|  | CL2 | 920 | 0.001 | 94.90 | 4.105 | 0.682 | HKY |
| 33-rpoB | F | 262 | 0.672 | 0.162 | 1.987 | 0.650 | HKY+I |
|  | CL1 | 262 | 0.970 | 0.014 | - | 0.663 | F81 |
|  | CL2 | 262 | 0.001 | 94.90 | - | 0.682 | HKY |
| 34-sdhA | F | 223 | 0.606 | 0.233 | 3.054 | 0.633 | HKY+G |
|  | CL1 | 223 | 0.004 | 71.77 | 3.015 | 0.577 | K80 |
|  | CL2 | 223 | 0.823 | 0.032 | 13.19 | 0.598 | K80+I |
| 35-tpiA | F | 484 | 0.531 | 0.391 | 2.372 | 0.663 | HKY+G |
|  | CL1 | 484 | 0.827 | 0.112 | 3.676 | 0.656 | HKY |
|  | CL2 | 484 | 0.791 | 0.037 | 2.973 | 0.660 | HKY+I |
| 36-tpiA | F | 559 | 0.549 | 0.352 | 2.489 | 0.670 | HKY+G |
|  | CL1 | 559 | 0.814 | 0.121 | 4.005 | 0.660 | HKY |
|  | CL2 | 559 | 0.822 | 0.030 | 2.869 | 0.669 | HKY+I |
| 37-trpE | F |  | - | - | - | - | - |
|  | CL1 | 517 | 0.548 | 62.19 | 2.514 | 0.700 | F81 |
|  | CL2 |  | - | - | - | - | - |
| 38-uup | F |  | - | - | - | - | - |
|  | CL1 | 645 | 0.001 | 95.26 | 2.186 | 0.680 | HKY |
|  | CL2 | 645 | 0.822 | 0.027 | 2.615 | 0.662 | HKY+I |
