# Supplementary material for: Increased knowledge of Francisella genus diversity highlights the benefits of optimised DNA-based assays
Source: BMC Microbiol. 2012 Sep 25;12:220. doi: 10.1186/1471-2180-12-220 (PMC3575276; doi:10.1186/1471-2180-12-220)
Supplement: Additional file 4 — Table of single-marker results. Comparison of inferred single-gene topologies to the whole-genome topology with respect to RF distance degree of incongruence, difference in resolution, the proportion of misidentified strains and SH test of incongruence. To test alternative topologies for markers with missing sequences, the corresponding leaves were removed from the whole-genome tree. [file 1471-2180-12-220-S4.docx]

Additional File 4. Comparison of inferred single marker topologies to the whole-genome topology.

The RF distance was obtained in the Treedist software, the bipartitions in the alternative topology not present in the whole genome topology is denoted inc, while the opposite (i.e. difference in resolution between alternative and reference topologies) is denoted res, the proportion of mis-identified isolates is denoted PMII, both at the subspecies and clade level. The Shimodaira-Hasegawa test is denoted SH, where (**) and (*) denotes rejection of the null-hypothesis of congruence at the p = 0.01 and p = 0.05 levels, respectively. To test alternative topologies for markers with missing sequences, the corresponding leaves were removed from the whole-genome tree.

| Marker name | Pop. | RF | inc. | res. | PMII subsp | PMII clade | SH test |
| --- | --- | --- | --- | --- | --- | --- | --- |
| 01_16S | F | 0.617 | 0.308 | 0.735 | 0.459 | 0.000 | 0.067 |
|  | CL1 | 0.810 | 0.000 | 0.895 | 0.636 |  | 0.299 |
|  | CL2 | 0.333 | 0.000 | 0.500 | 0.077 |  | 0.487 |
| 02_16s+ItS+23s_1 | F | 0.617 | 0.308 | 0.735 | 0.703 | 0.000 | 0.035 (*) |
|  | CL1 | 0.810 | 0.000 | 0.895 | 0.545 |  | 0.045 (*) |
|  | CL2 | 0.333 | 0.000 | 0.500 | 0.077 |  | 0.375 |
| 03_16s+ItS+23s_2 | F | 0.532 | 0.154 | 0.676 | 0.135 | 0.000 | 0.069 |
|  | CL1 | 0.583 | 0.000 | 0.737 | 0.045 |  | 0.437 |
|  | CL2 | 0.467 | 0.200 | 0.600 | 0.077 |  | 0.083 |
| 04_16s+ItS+23s_3 | F | 0.500 | 0.143 | 0.647 | 0.378 | 0.000 | 0.141 |
|  | CL1 | 0.538 | 0.143 | 0.684 | 0.364 |  | 0.214 |
|  | CL2 | 0.429 | 0.000 | 0.600 | 0.000 |  | 0.278 |
| 05_aroA_2 | F | 0.520 | 0.250 | 0.647 | 0.270 | 0.000 | 0.004 (**) |
|  | CL1 | 0.583 | 0.000 | 0.737 | 0.455 |  | 0.487 |
|  | CL2 | 0.500 | 0.333 | 0.600 | 0.000 |  | 0.047 (*) |
| 06_atpA | F | 0.538 | 0.333 | 0.647 | 0.216 | 0.000 | 0.001 (**) |
|  | CL1 | 0.615 | 0.286 | 0.737 | 0.318 |  | 0.076 |
|  | CL2 | 0.529 | 0.429 | 0.600 | 0.077 |  | 0.008 (**) |
| 07_dnaA | F | 0.520 | 0.250 | 0.647 | 0.243 | 0.000 | 0.001 (**) |
|  | CL1 | 0.538 | 0.143 | 0.684 | 0.364 |  | 0.083 |
|  | CL2 | 0.571 | 0.250 | 0.700 | 0.385 |  | 0.138 |
| 08_fabH | F | 0.345 | 0.143 | 0.471 | 0.162 | 0.000 | 0.038 (*) |
|  | CL1 | 0.310 | 0.000 | 0.474 | 0.273 |  | 0.490 |
|  | CL2 | 0.500 | 0.333 | 0.600 | 0.000 |  | 0.059 |
| 09_fopA_1 | F | 0.472 | 0.263 | 0.588 | 0.243 | 0.000 | <0.001 (**) |
|  | CL1 | 0.407 | 0.000 | 0.579 | 0.273 |  | 0.017 (*) |
|  | CL2 | 0.625 | 0.500 | 0.700 | 0.077 |  | <0.001 (**) |
| 10_fopA_2 | F | 0.444 | 0.250 | 0.559 | 0.243 | 0.000 | <0.001 (**) |
|  | CL1 | 0.357 | 0.000 | 0.526 | 0.273 |  | 0.250 |
|  | CL2 | 0.625 | 0.500 | 0.700 | 0.077 |  | <0.001 (**) |
| 11_fopA-in | F | 0.660 | 0.385 | 0.765 | 0.405 | 0.000 | <0.001 (**) |
|  | CL1 | 0.652 | 0.000 | 0.789 | 0.364 |  | 0.068 |
|  | CL2 | 0.714 | 0.500 | 0.800 | 0.154 |  | <0.001 (**) |
| 12_fopA-out | F | 0.529 | 0.294 | 0.647 | 0.324 | 0.000 | <0.001 (**) |
|  | CL1 | 0.520 | 0.000 | 0.684 | 0.364 |  | 0.305 |
|  | CL2 | 0.750 | 0.667 | 0.800 | 0.308 |  | <0.001 (**) |
| 18_groEL | F | 0.509 | 0.316 | 0.618 | 0.135 | 0.000 | 0.027 (*) |
|  | CL1 | 0.615 | 0.286 | 0.737 | 0.136 |  | 0.219 |
|  | CL2 | 0.412 | 0.285 | 0.500 | 0.000 |  | 0.062 |
| 22_lpnA | F | 0.583 | 0.286 | 0.706 | 0.324 | 0.000 | 0.129 |
|  | CL1 | 0.520 | 0.000 | 0.684 | 0.182 |  | 0.519 |
|  | CL2 | 0.667 | 0.000 | 0.800 | 0.154 |  | 0.527 |
| 24_lpnB | F | 0.617 | 0.308 | 0.735 | 0.486 | 0.000 | 0.026 (*) |
|  | CL1 | 0.739 | 0.250 | 0.842 | 0.636 |  | 0.164 |
|  | CL2 | 0.571 | 0.250 | 0.700 | 0.154 |  | 0.157 |
| 25_mdh | F | 0.574 | 0.231 | 0.706 | 0.459 | 0.000 | 0.018 (*) |
|  | CL1 | 0.818 | 0.333 | 0.895 | 0.818 |  | 0.242 |
|  | CL2 | 0.333 | 0.000 | 0.500 | 0.000 |  | 0.321 |
| 26_mutS | F | 0.574 | 0.231 | 0.706 | 0.270 | 0.000 | 0.046 (*) |
|  | CL1 | 0.600 | 0.167 | 0.737 | 0.318 |  | 0.034 (*) |
|  | CL2 | 0.571 | 0.250 | 0.700 | 0.154 |  | 0.192 |
| 27_parC | F | 0.382 | 0.190 | 0.500 | 0.135 | 0.000 | 0.075 |
|  | CL1 | 0.333 | 0.091 | 0.474 | 0.182 |  | 0.140 |
|  | CL2 | 0.500 | 0.333 | 0.600 | 0.000 |  | 0.176 |
| 29_pgm_1 | F | 0.647 | 0.471 | 0.735 | 0.297 | 0.000 | <0.001 (**) |
|  | CL1 | 0.667 | 0.200 | 0.789 | 0.272 |  | 0.082 |
|  | CL2 | 0.500 | 0.333 | 0.600 | 0.000 |  | 0.011 (*) |
| 30_prfB | F | 0.569 | 0.353 | 0.676 | 0.243 | 0.000 | 0.011 (*) |
|  | CL1 | 0.600 | 0.167 | 0.737 | 0.273 |  | 0.106 |
|  | CL2 | 0.500 | 0.333 | 0.600 | 0.000 |  | 0.069 |
| 31_putA | F | 0.609 | 0.250 | 0.735 | 0.432 | 0.000 | 0.077 |
|  | CL1 | 0.667 | 0.200 | 0.789 | 0.273 |  | 0.096 |
|  | CL2 | 0.692 | 0.333 | 0.800 | 0.154 |  | 0.152 |
| 32_rpoA_1 | F | 0.609 | 0.250 | 0.735 | 0.486 | 0.000 | 0.115 |
|  | CL1 | 0.909 | 0.667 | 0.947 | 0.818 |  | 0.323 |
|  | CL2 | 0.333 | 0.000 | 0.500 | 0.000 |  | 0.057 |
| 33_rpoB | F | 0.756 | 0.286 | 0.853 | 0.865 | 0.053 | 0.019 (*) |
|  | CL1 | - | - | - | - |  | - |
|  | CL2 | 0.571 | 0.000 | 0.727 | 0.000 |  | 0.478 |
| 34_sdhA | F | 0.583 | 0.286 | 0.706 | 0.351 | 0.000 | 0.015 (*) |
|  | CL1 | 0.667 | 0.200 | 0.789 | 0.364 |  | 0.037 (*) |
|  | CL2 | 0.600 | 0.400 | 0.700 | 0.154 |  | 0.483 |
| 35_tpiA | F | 0.444 | 0.250 | 0.559 | 0.108 | 0.000 | 0.049 (*) |
|  | CL1 | 0.481 | 0.125 | 0.632 | 0.136 |  | 0.095 |
|  | CL2 | 0.294 | 0.143 | 0.400 | 0.000 |  | 0.243 |
| 36_tpiA_2 | F | 0.382 | 0.190 | 0.500 | 0.054 | 0.000 | 0.035 (*) |
|  | CL1 | 0.429 | 0.111 | 0.579 | 0.091 |  | 0.138 |
|  | CL2 | 0.294 | 0.143 | 0.400 | 0.000 |  | 0.236 |
| 37_trpE | F | 0.555 | 0.000 | 0.714 | 0.516 | 0.000 | 0.204 |
|  | CL1 | 0.739 | 0.250 | 0.842 | 0.545 |  | 0.149 |
|  | CL2 | 0.111 | 0.000 | 0.200 | 0.000 |  | 0.476 |
| 38_uup | F | - | - | - | - | - | - |
|  | CL1 | 0.481 | 0.125 | 0.632 | 0.091 |  | 0.072 |
|  | CL2 | 0.375 | 0.167 | 0.500 | 0.077 |  | 0.430 |
