# Supplementary material for: Increased knowledge of Francisella genus diversity highlights the benefits of optimised DNA-based assays
Source: BMC Microbiol. 2012 Sep 25;12:220. doi: 10.1186/1471-2180-12-220 (PMC3575276; doi:10.1186/1471-2180-12-220)
Supplement: Additional file 5 — Optimal set of marker partitions. Optimisation of the subset of two to seven marker-sequence topologies to minimise incongruences and difference in resolution compared to the whole-genome topology. The numbers show the percentage of each marker included in the optimal configurations. The proportion of strains misplaced in the tree, average bootstrap support of optimal topologies and the SH test of incongruence is also reported. The total number of global optima was calculated from the output of the heuristic search analyses. [file 1471-2180-12-220-S5.docx]

Additional File 5. Optimal set of two to seven combined markers.

Marker topologies are compared to the whole-genome topology. The numbers reflects the percentage of a marker included in all optimal configurations. Optimization on resolution is denoted res while congruence (i.e. bipartition matching) optimization is denoted inc. The Shimodaira-Hasegawa test is denoted SH, where (**) and (*) denotes rejection of the null-hypothesis of congruence at the p = 0.01 and p = 0.05 levels, respectively. The proportion of mis-classified isolates to the correct subspecies group is denoted Subsp. mis. The total number of optima is denoted Optima.

|  | MLST marker | 2 genes |  | 3 genes |  | 4 genes |  | 5 genes |  | 6 genes |  | 7 genes |  |
| --- | --- | --- | --- | --- | --- | --- | --- | --- | --- | --- | --- | --- | --- |
|  |  | res | inc | res | inc | res | inc | res | inc | res | inc | res | inc |
|  | 01_16S | 0 | 0 | 3.85 | 12.50 | 0 | 8.99 | 0 | 6.63 | 1.89 | 7.86 | 1.58 | 6.19 |
|  | 02_16s+ItS+23s_1 | 0 | 0 | 3.85 | 12.50 | 0 | 8.89 | 0 | 5.49 | 1.27 | 6.33 | 0 | 4.70 |
|  | 03_16s+ItS+23s_2 | 0 | 0 | 7.69 | 0 | 0 | 5.62 | 0 | 3.66 | 0 | 0 | 0 | 2.92 |
|  | 04_16s+ItS+23s_3 | 0 | 0 | 0 | 0 | 0 | 2.25 | 2.86 | 4.57 | 3.80 | 7.63 | 1.58 | 6.37 |
|  | 05_aroA_2 | 0 | 0 | 0 | 0 | 0 | 2.25 | 2.86 | 3.89 | 2.53 | 3.72 | 4.76 | 3.49 |
|  | 06_atpA | 0 | 0 | 0 | 0 | 0 | 0 | 0 | 0.46 | 0 | 1.69 | 0 | 1.27 |
|  | 07_dnaA | 0 | 0 | 0 | 0 | 0 | 0 | 0 | 0 | 0 | 0.51 | 1.58 | 1.11 |
|  | 08_fabH | 50.00 | 50.00 | 34.62 | 37.50 | 25.00 | 23.59 | 20.00 | 18.08 | 17.72 | 15.81 | 7.93 | 11.86 |
|  | 09_fopA_1 | 0 | 0 | 0 | 0 | 0 | 0 | 0 | 0 | 0 | 0 | 0 | 0 |
|  | 10_fopA_2 | 0 | 0 | 3.85 | 0 | 0 | 0 | 0 | 0 | 0 | 0 | 0 | 0 |
|  | 11_fopA-in | 0 | 0 | 0 | 0 | 0 | 0 | 0 | 0 | 0 | 0 | 0 | 0 |
|  | 12_fopA-out | 0 | 0 | 0 | 0 | 0 | 0 | 0 | 0 | 0 | 0 | 0 | 0 |
|  | 18_groEL | 0 | 0 | 0 | 0 | 0 | 0 | 2.86 | 2.52 | 0 | 0.01 | 7.93 | 3.89 |
|  | 22_lpnA | 0 | 0 | 0 | 0 | 0 | 1.12 | 5.71 | 1.83 | 5.69 | 2.87 | 6.34 | 3.13 |
|  | 24_lpnB | 0 | 0 | 7.69 | 0 | 25.00 | 5.62 | 20.00 | 6.64 | 17.72 | 7.19 | 14.28 | 5.62 |
|  | 25_mdh | 0 | 0 | 0 | 0 | 0 | 0 | 0 | 2.52 | 5.07 | 3.80 | 0 | 5.12 |
|  | 26_mutS | 0 | 0 | 3.85 | 0 | 0 | 0 | 0 | 1.83 | 1.89 | 0.01 | 14.28 | 3.21 |
|  | 27_parC | 0 | 0 | 3.85 | 0 | 25.00 | 3.37 | 17.14 | 5.03 | 14.56 | 5.07 | 9.52 | 4.74 |
|  | 29_pgm_1 | 0 | 0 | 0 | 0 | 0 | 0 | 0 | 0 | 0 | 0 | 0 | 0 |
|  | 30_prfB | 0 | 0 | 0 | 0 | 0 | 0 | 0 | 0 | 1.89 | 1.69 | 0 | 1.56 |
|  | 31_putA | 0 | 0 | 0 | 0 | 0 | 3.37 | 0 | 3.66 | 0.63 | 4.82 | 0 | 2.58 |
|  | 32_rpoA_1 | 0 | 0 | 0 | 0 | 0 | 3.37 | 0 | 3.43 | 1.89 | 3.30 | 0 | 2.18 |
|  | 33_rpoB | 0 | 0 | 0 | 0 | 0 | 4.49 | 2.86 | 5.95 | 1.89 | 6.00 | 0 | 4.68 |
|  | 34_sdhA | 0 | 0 | 0 | 0 | 0 | 0 | 2.86 | 2.52 | 3.16 | 4.40 | 3.17 | 3.71 |
|  | 35_tpiA | 50.00 | 50.00 | 26.92 | 37.50 | 25.00 | 26.97 | 20.00 | 21.28 | 17.09 | 21.80 | 14.28 | 14.16 |
|  | 36_tpiA_2 | 0 | 0 | 3.85 | 0 | 0 | 0 | 0 | 0 | 1.27 | 0 | 0 | 0 |
| Metric | RF | 0.133 | 0.133 | 0.133 | 0.133 | 0.097 | 0.097 | 0.097 | 0.097 | 0.097 | 0.097 | 0.079 | 0.079 |
| Metric | Incongruence | 0.000 | 0.000 | 0.036 | 0.000 | 0.000 | 0.000 | 0.000 | 0.000 | 0.000 | 0.000 | 0.000 | 0.000 |
| Metric | Resolution | 0.235 | 0.235 | 0.235 | 0.235 | 0.176 | 0.176 | 0.176 | 0.176 | 0.176 | 0.176 | 0.147 | 0.147 |
| Metric | Average bootstrap support | 86.12 (0.32) | 86.12 (0.32) | 88.26 (0.29) | 86.14 (0.32) | 90.43 (0.25) | 86.73 (0.32) | 89.24 (0.29) | 89.24 (0.29) | 91.84 (0.25) | 91.84 (0.25) | 90.04 (0.27) | 90.04 (0.27) |
| Metric | Subsp. mis. | 0.053 | 0.053 | 0.000 | 0.053 | 0.000 | 0.000 | 0.000 | 0.000 | 0.000 | 0.000 | 0.000 | 0.000 |
| Test | SH | 0.463 | 0.463 | 0.261 | 0.497 | 0.145 | 0.423 | 0.467 | 0.467 | 0.628 | 0.482 | 0.614 | 0.614 |
| No | Optima | 1 | 1 | 8 | 2 | 1 | 19 | 6 | 80 | 28 | 219 | 6 | 71659 |
